# Detection of *Listeria monocytogenes* in a patient with meningoencephalitis using next-generation sequencing: a case report

**DOI:** 10.1186/s12879-020-05447-z

**Published:** 2020-10-01

**Authors:** Zi-Wei Lan, Min-Jia Xiao, Yuan-lin Guan, Ya-Jing Zhan, Xiang-Qi Tang

**Affiliations:** 1 The Second Xiangya Hospital, Central South University, No.139 Middle Renmin Road, Changsha, 410011 Hunan China; 2 Hugobiotech Co., Ltd, No 1 Disheng East Road, Daxing District, Beijing, 100000 China

**Keywords:** *Listeria monocytogenes*, Meningoencephalitis, Cerebrospinal fluid, Next-generation sequencing

## Abstract

**Background:**

*Listeria monocytogenes* (*L. monocytogenes*) is a facultative intracellular bacterial pathogen which can invade different mammalian cells and reach to the central nervous system (CNS), leading to meningoencephalitis and brain abscesses. In the diagnosis of *L. monocytogenes* meningoencephalitis (LMM), the traditional test often reports negative owing to the antibiotic treatment or a low number of bacteria in the cerebrospinal fluid. To date, timely diagnosis and accurate treatment remains a challenge for patients with listeria infections.

**Case presentation:**

We present the case of a 66-year-old woman whose clinical manifestations were suspected as tuberculous meningoencephalitis, but the case was finally properly diagnosed as LMM by next-generation sequencing (NGS). The patient was successfully treated using a combined antibacterial therapy, comprising ampicillin and trimethoprim-sulfamethoxazole.

**Conclusion:**

To improve the sensitivity of LMM diagnosis, we used NGS for the detection of *L. monocytogenes*. Hence, the clinical utility of this approach can be very helpful since it provides quickly and trust results.

## Background

*Listeria monocytogenes* (*L. monocytogenes*) is a Gram-positive bacillus, its genetic machinery allows bacterial life as a facultative intracellular pathogen. *L. monocytogenes* widely exists in the environment at high salt concentrations and fairly low moisture content [1]. It is well adapted to survive and persist in food processing facilities, entering the final products (such as ready-to-eat) by cross-contamination. Additionally, this organism is also psychrophilic and has a competitive advantage over other Gram-positive and Gram-negative microorganisms in cold environments, such as refrigerators [2]. The consumption of contaminated foods is considered the main cause leading of listeriosis.

Invasive forms of listeriosis can lead to infection of CNS, in these cases even though the adequate antibiotic treatment the overall mortality rates remains nearly 30% [3]. The clinical manifestations of LMM are usually uncharacteristic, thus hindering prompt diagnosis [4]. Due to adequate antibiotic therapy or low number of bacteria, traditional approaches often fail to detect *L. monocytogenes* in the cerebrospinal fluid (CSF) [5]. Therefore, to enhance the diagnostic accuracy of LMM, an unbiased diagnostic approach is necessary. Next-generation sequencing (NGS) is a comprehensive and equitable pathogen detection tool, with the potential to improve pathogen identification [6]. Unlike traditional testing, NGS can identify novel or unexpected pathogens in the CSF or brain tissue screens for nearly all potential CNS infections.

To date, only a few studies have evaluated the use of NGS for the clinical diagnosis of LMM. Herein, We report the use of NGS as a helpful diagnostic method, which enabled the identification of LMM in this case, leading to proper treatment and fully recovery of the patient. To our knowledge, this is the first reported case in which the patient was initially misdiagnosed as tuberculous meningoencephalitis and finally diagnosed as LMM by NGS.

## Case presentation

A 66-year-old formerly healthy woman was admitted to the hospital with a 4-day history of headache, fever, nausea, and vomiting, followed by changes in consciousness. On admission, she presented with neck stiffness, positive Kernig sign, and a Glasgow Coma Scale (GCS) score of 12. A lumbar puncture (LP) was performed: the CSF was canary yellow, the opening pressure was 250 mmH_2_O, with 580 × 10^6^/L white cells (neutrophils 14%, lymphocytes 69%, large lymphocytes 5%, monocytes 12%). The protein level was significantly elevated (978 mg/L), while the CSF glucose level (2.06 mmol/L, blood glucose 7.3 mmol/L) and chloride level (110.8 mmol/L) were decreased. Gram stain, acid-fast stain, and ink stain of the CSF were negative. Additionally, CSF and blood cultures were negative. Laboratory examination suggested hyponatremia (129 mmol/L). Immunological screenings, including an evaluation of humoral immunity (immunoglobulin levels and subclass IgG), cellular immunity (total and subpopulations of T cells), and complement (C3 and C4), were normal. The tumor-related screening was normal. Tests for human immunodeficiency virus (HIV) and infectious hepatitis were negative. Brain computed tomography (CT) and magnetic resonance imaging (MRI) indicated no obvious abnormalities. Therefore, the patient was diagnosed with tuberculous meningoencephalitis. Following the administration of anti-tubercular therapy (ATT), the headache and fever were relieved. On reexamination, LP improved but remained abnormal. Three days later, the NGS of CSF was suggestive of *Listeria*, and we immediately amended the therapeutic regimen to ampicillin plus trimethoprim-sulfamethoxazole (TMP-SMX). After 4 weeks, the headache, and fever resolved, and the LP results were completely normal.

The patient originated from China and lived in Changde, Hunan province, since birth. She was a housewife without recent travel experience and denied eating raw or semi-raw food, or unpasteurized dairy products. She had no history of autoimmune disease and did not use tobacco or illicit drugs.

### Sample collection and processing

Approximately 2–3 mL of CSF was collected and sealed using a sterile technique, shipped on dry ice to Hugobiotech Co., Ltd. (Beijing, China) to perform NGS detection. The DNA was extracted and purified from 200 μL of CSF supernatant according to the manufacturer’s instructions for the TIANGEN DNA Mini kit DP316 (Tiangen Biotech, Beijing, China). The DNA libraries were constructed via end-repaired adaptation and application of polymerase chain reaction (PCR). The concentration and quality of the libraries were evaluated using Qubit 1*dsDNA HS Assay Kit (Invitrogen, USA) and agarose gel electrophoresis (Sangon Biotech, Shanghai, China). Qualified libraries with different barcode labeling were pooled together, and then sequenced on an Illumina NexSeq platform (Illumina, San Diego, CA).

### Data analysis

**Fig. 1:**
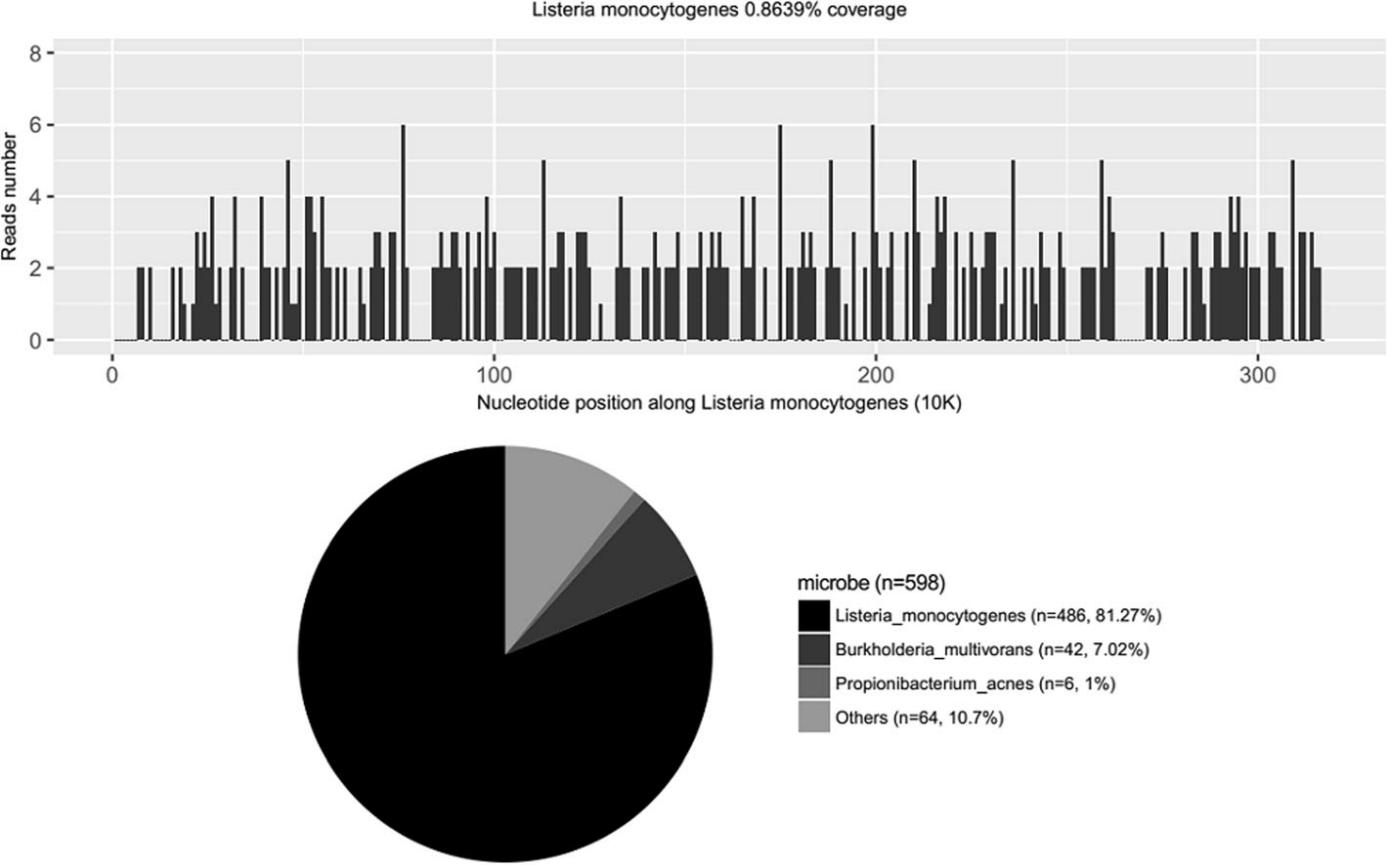
NGS results of pathogen identification: 81.27% of bacterial reads corresponded to *L. monocytogenes* with a coverage of 0.8693%. *L. monocytogenes*: *Listeria monocytogenes*, NGS: next-generation sequencing

After obtaining the sequencing data, high-quality data were generated after filtering out adapter, low-quality, low-complexity, and shorter reads. Next, human reads were removed by mapping reads to human reference genome (GRCh38 sequences) using Scalable Nucleotide Alignment Program software (1.0beta.18, Matei Zaharia, 2015). The remaining data were aligned to the microbial genome database using the Burrows-Wheeler Alignment (version 0.7.15-r1140, Li.2016). The database collected microbial genomes from the National Center for Biotechnology Information, and contained more than 20,000 microorganisms, including 11,910 bacteria, 7103 viruses, 1046 fungi, and 305 parasites. The alignment results were used to calculate the coverage and depth of each species calculated. The number of sequencing reads was 5,942,851. Of these, 486 reads uniquely aligned to the *L. monocytogenes* genome. After excluded the reads from human host, *L. monocytogenes* reads show dominant abundance in all microbial species, with a percentage of 81.27% (Fig. 1).

## Discussion and conclusion

China is one of the greatest contributors to the global tuberculosis burden, with a high prevalence rate of smear-positive tuberculosis, approximating 59/100,000 [7, 8]. According to the clinical scoring system for tuberculous meningitis, this patient scored more than 6 points [9]. Combined with the clinical score results and epidemiological data, the patient was diagnosed with tuberculous meningoencephalitis, and hence ATT was initiated. Following the results of NGS, the therapeutic regimen was immediately modified, and the patient finally recovered. In China, listeriosis is currently not listed as a legal infectious disease. Although the incidence of listeriosis has increased in recent years, it is still sporadic. Records on patients with listeriosis reported from 2011 to 2017 in China were only 698 [4, 10]. As elderly people in China prefer to eat well-cooked food instead of raw or semi-raw food, compared with foreign countries, the mean age of patients infected with *Listeria* demonstrated younger demographic [4]. The clinical symptoms and CSF of LMM are similar to those of other meningoencephalitis. Most patients present a subacute course, and almost all patients demonstrate at least two of the following four symptoms, including fever, headache, neck stiffness, and a change in mental status [11]. Previously, nystagmus and abducen nerve palsy have also been reported as initial symptoms [12]. The CSF of LMM generally indicated an increase in the number of white blood cells with prominent lymphocytes (> 25%), accompanied with an increase in protein concentration and low CSF glucose. Approximately one-third of the patients demonstrated a positive Gram stain.

The timely identification of pathogens and the initiation of appropriate antibiotic treatment is the key for a favorable prognosis. Methods for pathogen discovery, such as bacterial culture, have existed for years and remain the gold standard for the diagnosis of listeria infections, however, these methods are time-consuming, cumbersome and lack sensitivity. In addition, the positive rate of CSF culture is low (around 40%) [13]. Molecular techniques such as specific polymerase chain reaction (PCR) have been recommended for diagnosing encephalitis caused by the herpes simplex virus, varicella-zoster virus, and enterovirus [14, 15]. However, the application of PCR in CNS listeriosis has not been popularized in China, and only mentioned in a few case reports [16, 17]. Although the sample-to-answer turn-around time of PCR is often less than 12 h, it relies on the prior knowledge of the causative agent. Specific PCR does not allow the identification of unexpected or novel pathogens and has difficulties in testing various rare pathogens [18]. NGS overcomes the limitations of targeted molecular diagnostic methods, as prior knowledge or assumptions regarding the infection inducing pathogens are not essential [19, 20]. Theoretically, NGS can identify almost all microorganisms based on specific nucleic acid sequences, with adequately long reads, multiple hits in the microbial genome, and a complete reference database. NGS has a higher sensitivity for pathogen identification and is less affected by prior antibiotic usage. NGS has been successfully employed for pathogen detection in CSF. A prospective multicenter study has demonstrated that NGS was more effective and rapid for detection of CNS infection than conventional gold standard methods [5]. With the rapid development of sequencing speeds, the sample-to-answer turn-around time of NGS can be shortened to 48 h, providing patients with a more accurate treatment strategy [18].

The combination of ampicillin and TMP-SMX has previously been described as an effective treatment for CNS listeriosis [21, 22]. A retrospective case-control study indicated that delaying appropriate antibiotic therapy for more than 6 h increased the risk of mortality by 2.78-fold [23]. Therefore, an early diagnosis is crucial, followed by timely treatment. Several in vitro studies have demonstrated the antibacterial activity of rifampicin against *L. monocytogenes*, which could explain the improvements observed in the patient’s headache and CSF results during ATT [1, 24]. Furthermore, the previous misdiagnosis of patients with LMM as drug-resistant tuberculous meningoencephalitis, prior to NGS, needs to be addressed. Early use of dexamethasone is recommended for other types of bacterial meningoencephalitis. However, dexamethasone can result in disease progression and increase mortality in CNS listeriosis [25, 26]. Furthermore, treatment duration of LMM needs to be maintained for at least 3 weeks or continued until brain imaging confirms the disappearance of lesions [27].

A Danish cohort study reported that patients with listeria encephalitis presented a significantly higher mortality rate than the general population within 5 years of diagnosis, primarily due to cancer [28]. To improve the survival rate, patients with CNS listeriosis should be meticulously screened for underlying malignant diseases.

Due to the non-specific CSF findings and low positivity rate of culture, the early diagnosis of LMM remains a clinical challenge. This case highlights the feasibility of using NGS as a early diagnostic assay for CNS listeriosis, and proposes that in patients with clinically suspected tuberculous meningoencephalitis, the possibility of Listeria infection needs to be considered.

## Data Availability

The data that support the findings of this study are available from the corresponding author (XQT and YJZ) upon reasonable request.

## Abbreviations

ATT: Anti-tubercular therapy
CNS: Central nervous system
CSF: Cerebrospinal fluid
CT: Computed tomography
GCS: Glasgow Coma Scale
HIV: Human immunodeficiency virus
*L. monocytogenes*: *Listeria monocytogenes*
LMM: *Listeria monocytogenes* meningoencephalitis
LP: Lumbar puncture
MRI: Magnetic resonance imaging
NGS: Next-generation sequencing
PCR: Polymerase chain reaction
TMP-SMX: Trimethoprim-sulfamethoxazole
